# Supplementary material for: Silencing of Putative Plasmodesmata-Associated Genes PDLP and SRC2 Reveals Their Differential Involvement during Plant Infection with Cucumber Mosaic Virus
Source: Plants (Basel). 2025 Feb 6;14(3):495. doi: 10.3390/plants14030495 (PMC11819965; doi:10.3390/plants14030495)
Supplement: Supplementary file 1 [file plants-14-00495-s001.zip › plants-3314833-supplementary.pdf]

Supplementary material

Figure legends

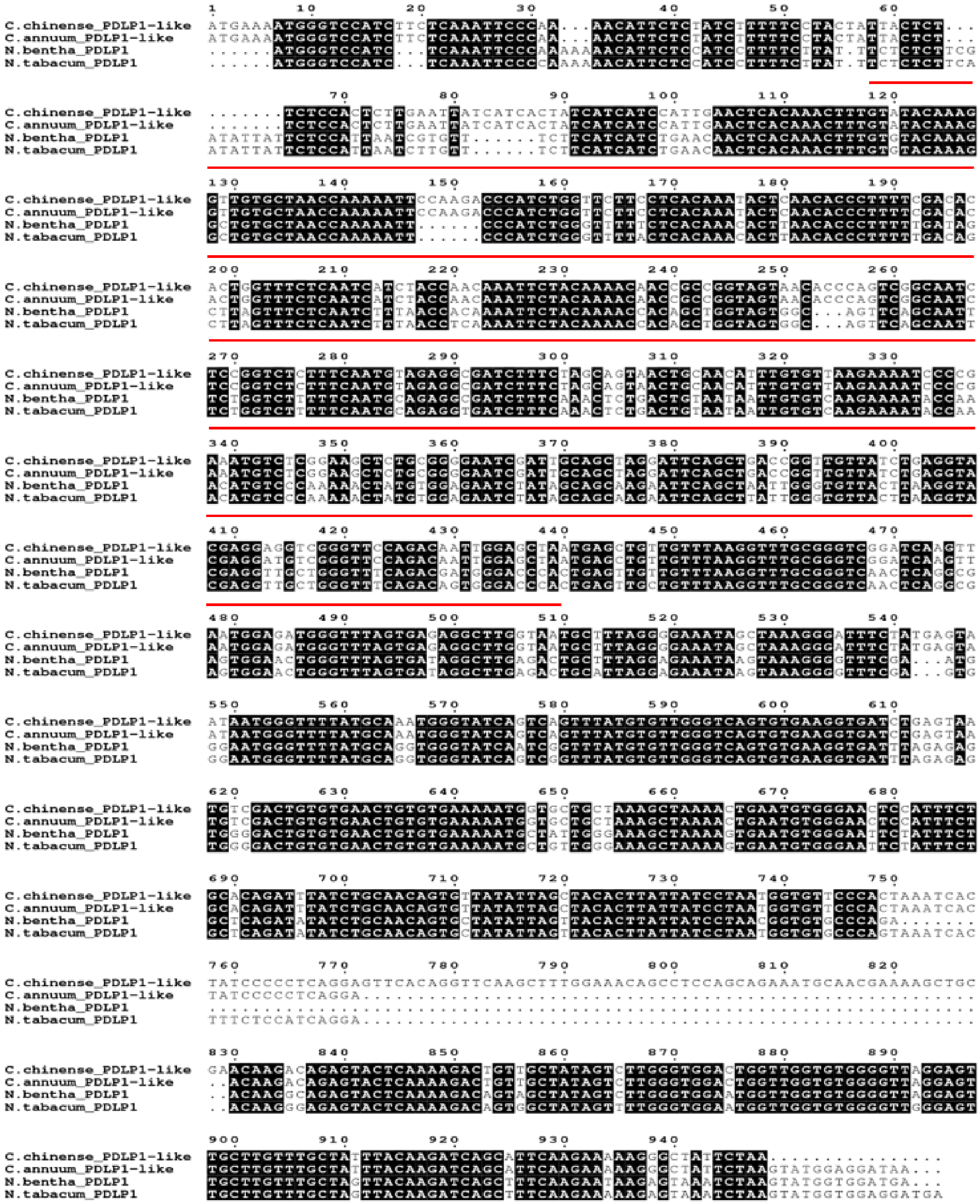

Figure S1. Multiple sequence alignment of *PDLP* genes of *Nicotiana benthamiana* (Niben101Scf03456g00018.1), *Capsicum chinense* (MCIT02000006.1), *C. annum* (XM_016721975.2), and *N. tabacum* (XM_016610375.1). Red colored lines indicate gene fragment (383 bp) used as VIGS targeted region for silencing of the *PDLP* gene. Sequence alignment was performed using Clustal Omega.

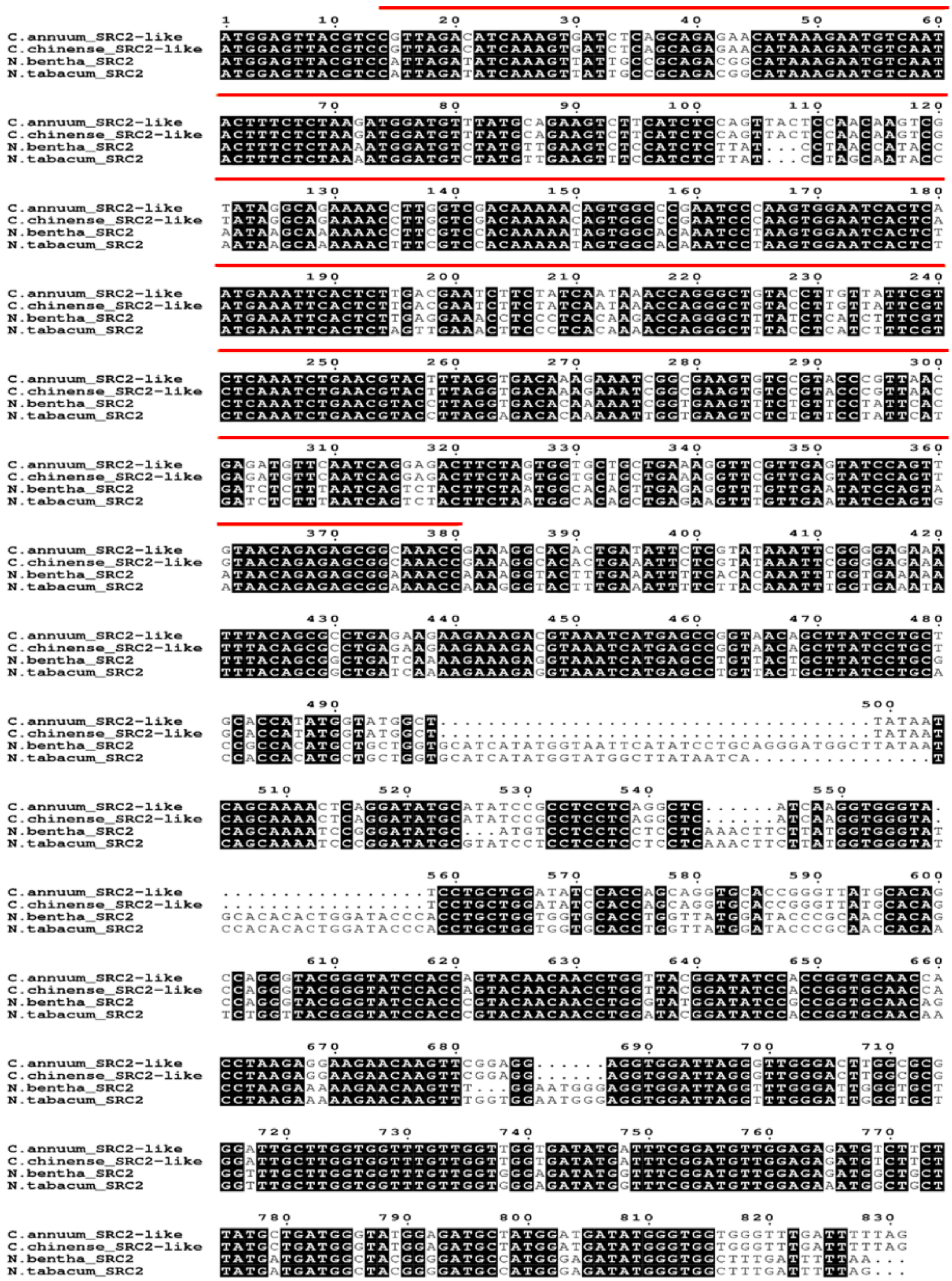

Figure S2. Multiple sequence alignment of *SRC2* genes of *Nicotiana benthamiana* (DQ465395.1), *Capsicum chinense* (AB442165.1), *C. annum* (NM_001324570.1) and *N. tabacum* (XM_016630487.1). Red colored lines indicate gene fragment (364 bp) used as VIGS targeted region for silencing of the *SRC2* gene. Sequence alignment was performed using Clustal Omega.

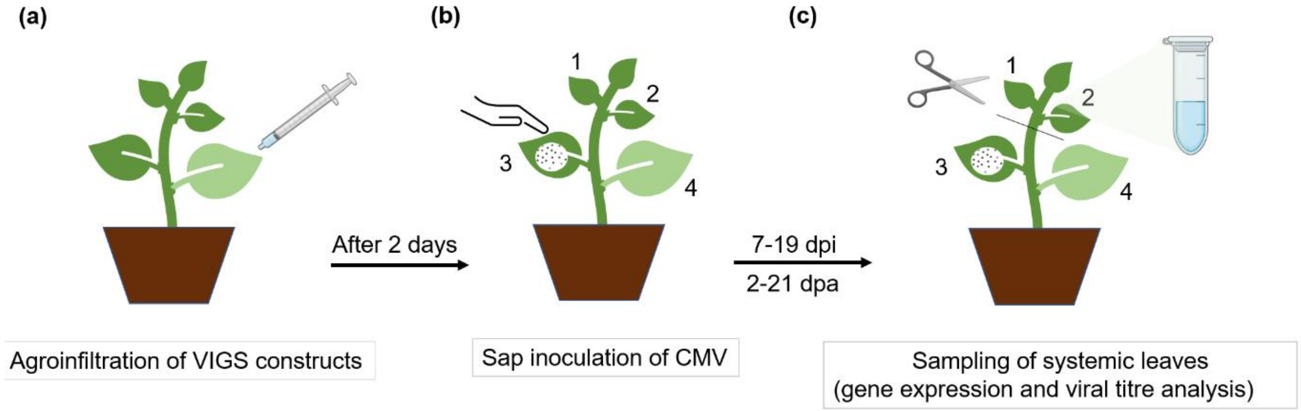

Figure S3. Illustrative depiction of the methodology involved in the agroinfiltration of the VIGS constructs, cucumber mosaic virus (CMV) inoculation, and gene expression analysis in the experimental plants.

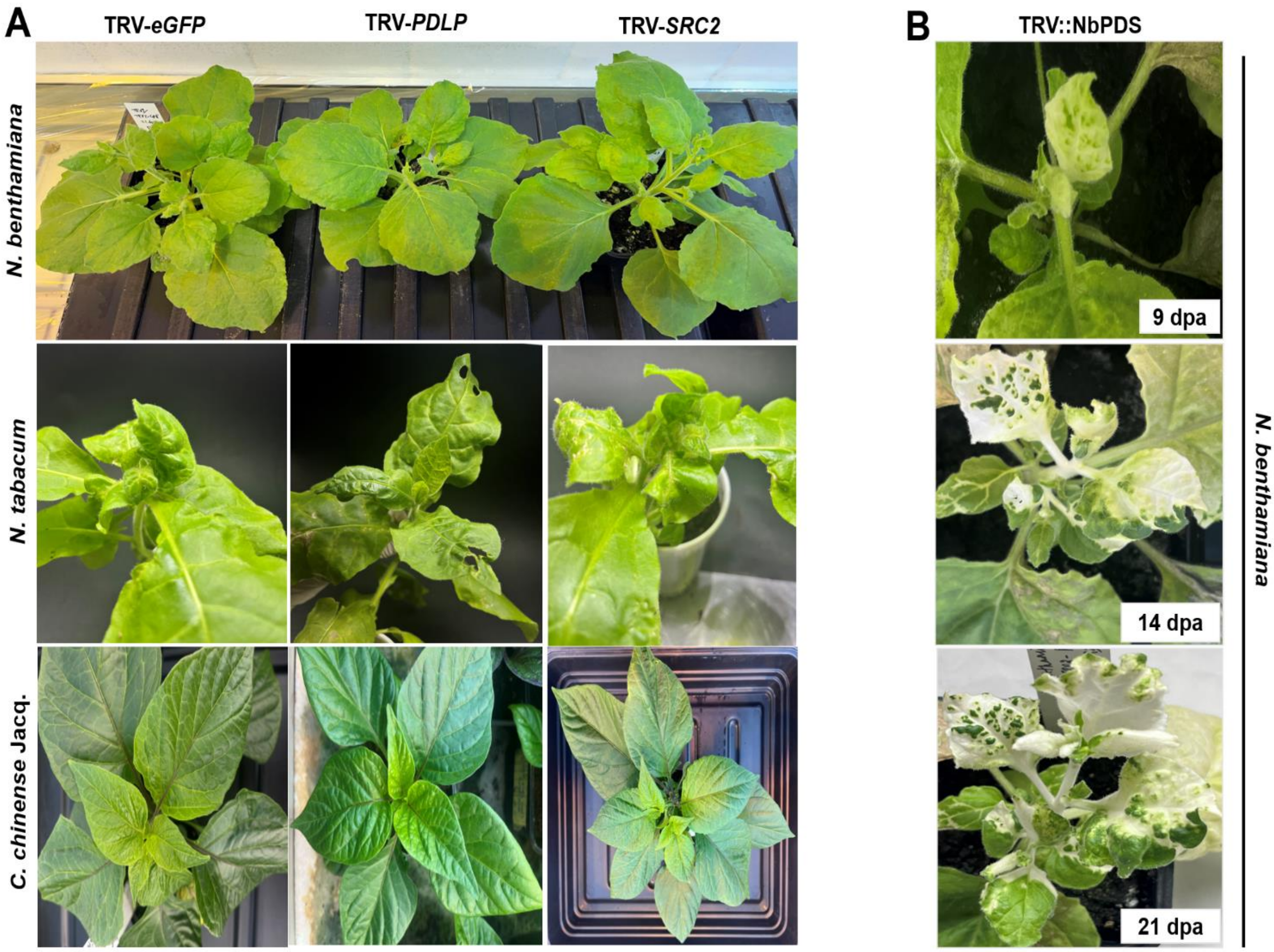

Figure S4. Phenotypic observations of VIGS silenced *Nicotiana benthamiana*, *Nicotiana tabacum*, and *Capsicum chinense* plants (A) at 21 days post agroinoculation (dpa) and (B) Phytoene desaturase *(PDS)* gene was used as a positive control. Silencing of *PDLP* and *SRC2* genes did not alter the development in the *N. benthamiana* and *C. chinense*; in contrast *N. tabacum* exhibited some leaf crumbling symptoms.

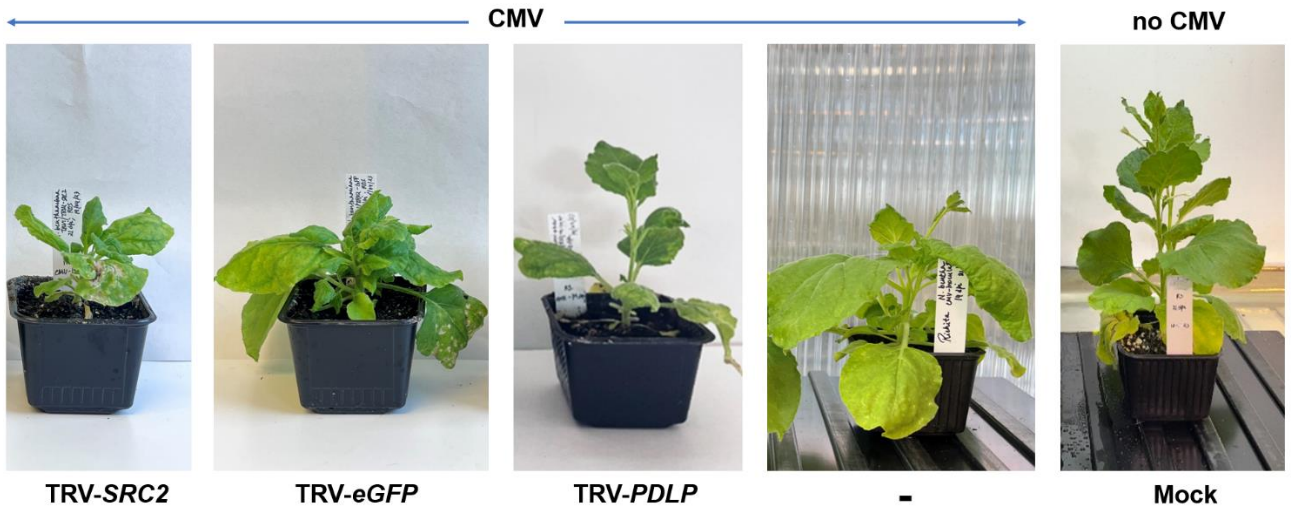

Figure S5. Representative images of *N. benthamiana* plants upon CMV-inoculation at 19 dpi. dpi= days post CMV inoculation, CMV= cucumber mosaic virus, (**-**) = non silenced plant with CMV infection, Mock= Phosphate buffer treated plant. TRV-*SRC2*, TRV-*eGFP*(control), and TRV-*PDLP* are VIGS silenced plants observed upon CMV inoculation.

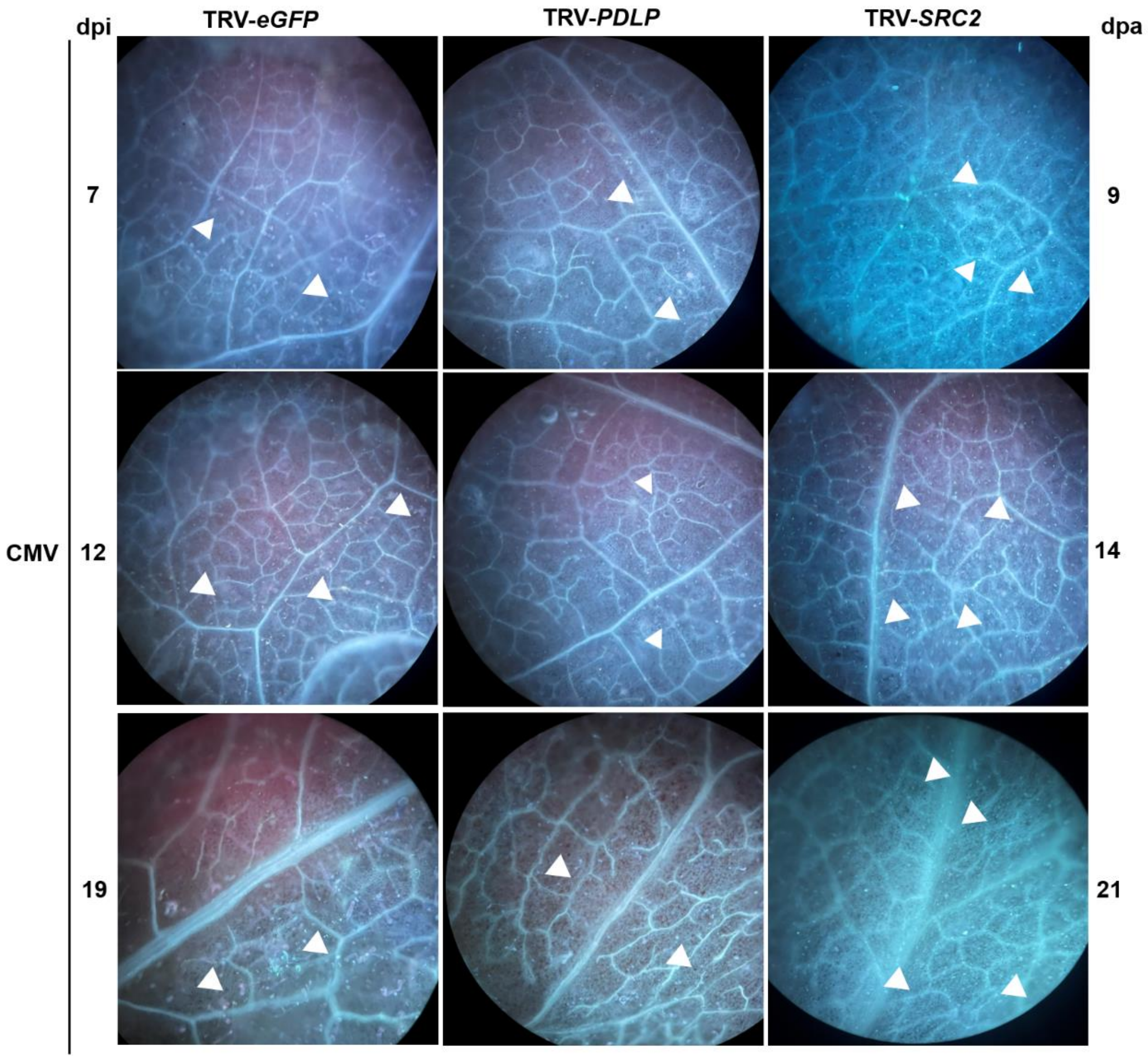

Figure S6. Microscopic observation of callose deposits upon cucumber mosaic virus (CMV) in *PDLP* and *SRC2* silenced *Nicotiana benthamiana* plants. Callose deposition assay using aniline blue staining was performed according to the procedure described by Schenk et al. 2015. The callose deposition was visualized using a fluorescent microscope with UV lamp. White arrow indicates callose deposits. dpa=days post agroinoculation. dpi=days post infection.

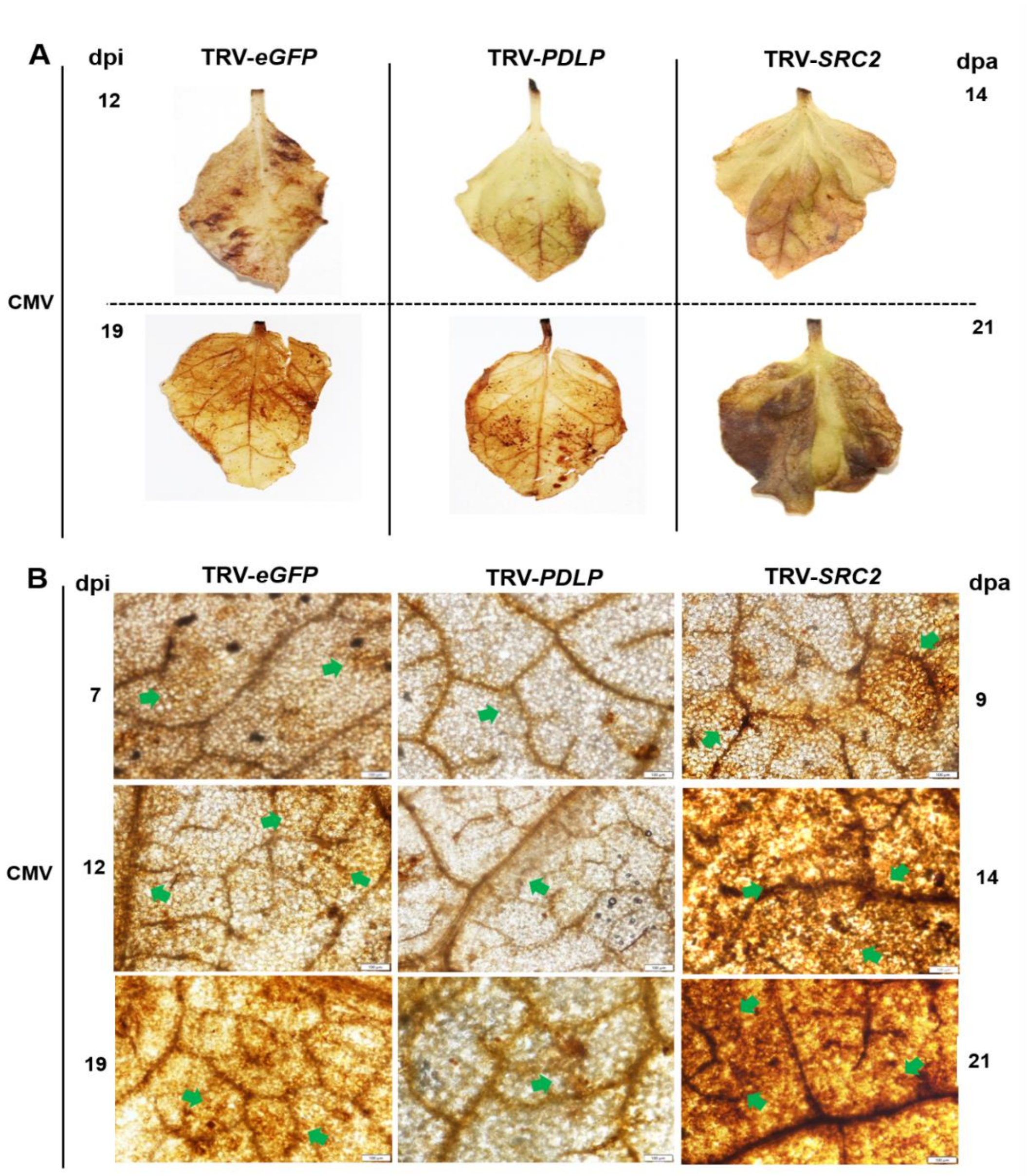

Figure S7. Macroscopic (A) and microscopic (B) detection of H_2O_2 by 3,3- diaminobenzidine (DAB) staining in VIGS-silenced *Nicotiana benthamiana* plants at different time-points after DAB staining. Control (*eGFP*) and *SRC2*-silenced plants exhibited elevated levels of H_2O_2 as compared to the *PDLP*-silenced group. Dark brown patches (indicated by green arrows) represent areas of reactive oxygen species (ROS) accumulation; (see results of RT-qPCR in Fig. 7), where darker shades indicate higher viral accumulation and lighter shades indicate lower viral accumulation. DAB assay was performed following the procedure outlined by Bach-Pages and Preston 2018. dpa=days post agroinoculation; dpi=days post CMV infection.
